# Anthropometric and Somatotype Profile of Elite Finn Class Sailors

**DOI:** 10.3390/jfmk9030121

**Published:** 2024-07-05

**Authors:** Luka Pezelj, Boris Milavić, Mirjana Milić

**Affiliations:** 1 Faculty of Maritime Studies, University of Split, 21000 Split, Croatia; 2 Faculty of Kinesiology, University of Split, 21000 Split, Croatia; mirjana.milic@kifst.hr

**Keywords:** dinghy sailing, elite athletes, Finn class, fitness testing, morphological characteristics, Olympic sailing, sailing, somatotype

## Abstract

Determining the reference base of anthropometric parameters on a sample of elite athletes is one of the foundations of further research and forming a clearer picture of each sport and sports discipline. In this study, the aim was to describe the anthropometric and somatotype profiles of elite Finn class sailors and to determine the differences in the measured parameters between sailors at different levels of general competitive success. The subject sample included 57 Finn class sailors who competed at the open Finn European Championship. A set of 25 anthropometric variables were applied. The sailors were divided into three groups according to their level of general competitive success using World Sailing Rankings. Finn sailors had higher average values in almost all morphological characteristics when compared to the sailors in other Olympic classes. Considering the average values of somatotype categories, we determined that Finn sailors fit the *endomorphic mesomorph* somatotype category (3.94 ± 1.19 − 5.50 ± 1.19 − 1.63 ± 0.74). Significant differences were observed between more-successful, medium, and less-successful sailors in the variables of *age*, *body mass*, *muscle mass*, *arm muscle mass*, and *endomorphy rating*. These results indicate the possibility of selection processes and/or adaptation to sailing occurring in the Finn class. The anthropometric characteristics of Finn sailors compared to sailors in Olympic classes further “support” the Finn class being called the “heavy dinghy” male class. This study on anthropometric parameters, determined via a sample of top Finn sailors, may be of great help to coaches and young sailors when deciding on the selection of an adult sailing class.

## 1. Introduction

Determining the reference base of anthropometric parameters on a sample of elite athletes is one of the foundations of further research and forming a clearer picture of each sport and sports discipline. Somatotype, the quantification of the present shape and composition of the human body calculated from the values of anthropometric parameters, provides an additional dimension that is useful in understanding and interpreting the anthropometric profile of elite athletes. It is expressed as representing relative fatness as an endomorphy rating, musculoskeletal robustness as a mesomorphy rating, and relative linearity of a physique as an ectomorphy rating [1]. Relations of amount and relations of muscle and fat mass according to a somatotype rating report a high positive correlation between endomorphy and percentage of body fat, a low positive correlation between mesomorphy and fat free weight, and a low know negative correlation between ectomorphy and percentage of body fat and fat-free weight [2,3].

Reference bases should be up to date as anthropometric characteristics are constantly evolving as a response to changes in the sporting and external environment [4]. Reference bases for anthropometric parameters and the somatotype on a sample of elite athletes have been determined for numerous sports [5,6,7,8,9,10], as well as the impact of some anthropometric characteristic [5,8,11,12,13,14,15] and somatotype ratings on competitive success [7,8,12,14,16]. Higher lean mass does appear to benefit sprint athletes [5] and sprint cross-country skiers [13], lower values of body mass index and percentage of body fat benefit elite mountain bikers [8], and the performance of the sailors in the Laser class, among other parameters, are determined by height and sitting height [11]. Results from somatotype rating research present significantly lower values for the endomorphic component in more-successful mountain bikers [8] and windsurfers [12].

Sailing is a sport in which athletes have the opportunity to compete in many sailing classes. The most competitive, i.e., the most “sportlike”, sailing classes are those included in the Olympic program, the so-called Olympic classes. The Finn class was the longest-serving Olympic class as it had been included in the Olympic program from 1952 to Tokyo 2020, and the adjective accompanying its name (heavy-weight dinghy) suggests that it is a class intended for the “biggest” of sailors.

The selection of a sailing class that allows a sailor to use his or her potential to the fullest is one of the most important steps in every sailor’s sports career. One of the criteria for the selection of a sailing class is the compatibility of the anthropometric profile of the sailor with the technical specifications and other specificities of sailing in each sailing class. Coaches and athletes are familiar with some anthropometric parameters from their experience and mutual exchange of data; however, scientific studies with standardized measurements and valid data are substantially lacking. There are papers which do not even focus their research on anthropometric parameters but still offer useful data on the anthropometric parameters of stature and body mass for sailors in some sailing classes [17,18,19,20,21,22,23,24,25,26]; however, more detailed studies, including a greater number of anthropometric parameters and (or) somatotype, are considerably less common.

This study aimed to describe the anthropometric and somatotype profile of elite former Olympic Finn class sailors and to determine the differences in the measured parameters between sailors at different levels of general competitive success.

## 2. Materials and Methods

### 2.1. Participants

The subject sample included 57 elite Olympic Finn class sailors who competed at the Open Finn European Championship (FEC), held 9–17 May 2015, in Split, Croatia. The FEC is an open type of competition; thus, apart from the European sailors, it also included the best-ranked world sailors, among which were Olympic, world, and continental medal-winners. The competition included 70 sailors; therefore, the 57 sailors who participated in the study represent 81% of the total number of participants in the competition.

All sailors participated in the study voluntarily. The study was approved by the Scientific Committee of the Faculty of Kinesiology in Split, was conducted with the support of the Executive Committee of the International Finn Association, and met the requirements of the Declaration of Helsinki (1964) and the ethical standards in sports and exercise research.

### 2.2. Measures

A set of anthropometric variables measured by anthropometric measuring tools was applied—stature, sitting height, biepicondylar humerus width, biepicondylar femur width, upper arm girth (flexed and tensed), calf girth, triceps skinfold, subscapular skinfold, supraspinale skinfold, and medial calf skinfold—from which we subsequently calculated body mass index, sum of skinfolds, and somatotype following the Heat–Carter method [1]. Cut-off values for the endomorphy rating were set from 0.5 to 16, for the mesomorphy rating from 0.5 to 12, and for the ectomorphy rating from 0.5 to 9 [27].

All measurements were conducted following the International Society for the Advancement of Kinanthropometry (ISAK) protocol [28] on the dominant side of the body, as suggested in the original instructions for using the Heath–Carter method for somatotype calculation [1]. Moreover, the subjects were measured by using the Tanita BC-418 (Tanita Corp., Tokyo, Japan) device, which uses a constant current source with a high frequency current (50 kHz, 90 µA), following the recommendations given by Kyle et al. [29]. The method of bioelectric impedance was used to determine the results of the following morphological measures: *body mass*, *fat range*, *muscle mass*, *trunk muscle mass*, *arms muscle mass*, *legs muscle mass*, *fat mass*, *trunk fat mass*, *arms fat mass*, and *legs fat mass*. The subjects took the BIA measurement barefoot and only in dry underwear. All jewelry, watches, or any other pieces of clothing were taken off. The GMON software was used to conduct measurements where the “body type” value was set for all subjects to “sports mode” and the “clothing weight” value was set to 0.0 kg.

Considering that sailing experience can be an important factor of success in sailing, in addition to the previously mentioned sets of variables, we also applied the *age* variable.

### 2.3. Procedures

This study was designed as a single cross-sectional study.

The same morphologically expert measurer conducted all the measurements in the week before the competition in the morning hours before the first training session. Every measurement per sailor was conducted in a maximum time of 30 min.

The sailors were divided into three groups according to their level of general competitive success: more successful (1), medium (2), and less successful (3). We determined the general success criterion via their ranking in the World Sailing Rankings (WSR). The WSR is formed by collecting the points from the six most successful competitions for each sailor in the 12 months from the publishing of the table. For this study, the WSR table for the Finn class published on 27 April 2015—the last one before the FEC started—was used. The group of sailors with a higher level of general success (1) included the subjects ranked among the first 20 sailors according to the WSR; the group of sailors with a medium level of general success (2) included the subjects ranked from the 20th to the 40th place of the WSR; and the group of sailors with a lower level of general success (3) included the subjects ranked lower than the 41st place of the WSR.

### 2.4. Statistical Analysis

Methods of data analysis included the calculation of basic statistical indicators—mean, standard deviation, minimum result, maximum result—and the determination of the measures of sensitivity of result distribution: skewness, kurtosis, maximum distance between relative cumulative theoretical frequency (normal), and relative cumulative empirical frequency (obtained by measuring). The results of the Kolmogorov–Smirnov test of the observed variables indicate that neither of the variables exceeds the cut-off value of the Kolmogorov–Smirnov test, which is 0.18 for the observed sample. These findings indicate that the variables do not deviate significantly from the normal distribution, and all variables are suitable for further parametric statistical analysis. The differences between groups of sailors were determined via one-way analysis of variance (ANOVA). Further, post hoc analyses of differences between the groups of Finn sailors were made using Fisher’s LSD test. To determine effect size of the differences found, squared eta (η^2^) coefficients were calculated and interpreted according to the criterion of Gamst et al. (2008) [30].

Data analysis was performed by using the STATISTICA software package (ver. 14.00).

## 3. Results

Table 1 presents descriptive indicators of all the measured variables: arithmetic mean, standard deviation, median, and minimum and maximum result. We conducted the analysis of sensitivity based on coefficients of asymmetry and peakedness of distribution, whereas we used the Kolmogorov–Smirnov test to test the normality of distribution.

Coefficients of asymmetry for the variables *legs fat mass*, *subscapular skinfold*, and *medial calf skinfold* indicate a slightly positive asymmetry, whereas the *calf girth* variable has a slight negative skew. Coefficients of peakedness indicate a somewhat lower sensitivity of the *legs fat mass* and *calf girth* variables.

Table 2 presents the descriptive parameters (arithmetic means and standard deviations) results of the univariate analysis of differences (ANOVA) (coefficient of analysis of variance and significance of differences) and the results of the post hoc analysis of differences conducted via the Fisher’s LSD test (significance of differences).

By applying univariate analysis of differences, significant differences were found in the results of the arithmetic means for the variables *age* and *body mass* between the groups of elite sailors according to the general competitive success criterion.

Through post hoc analysis of differences, significant differences were found between groups of sailors at different levels of general competitive success for the variables *age* (higher vs. lower; medium vs. lower), *body mass* (higher vs. lower; medium vs. lower), *muscle mass* (higher vs. lower), *arms muscle mass* (medium vs. lower), and *endomorphy rating* (higher vs. medium).

The effect size [30] of these differences was high for the variable *age*, moderate for the variable *body mass*, and low for the other variables (*muscle mass*, *arms muscle mass*, and *endomorphy rating*).

Table 3 presents the classification of elite Finn sailors according to the somatotype category. The frequency and percentage of each somatotype category was calculated for the total sample.

The analysis in Table 3 shows that out of the 13 possible somatotype categories, elite Finn sailors fit 7 categories. Just over 80% of the total sample of elite sailors fit the somatotype categories with the dominant *mesomorphic* component, 68.42% of which fit the *endomorphic mesomorph* category.

**Figure 1:**
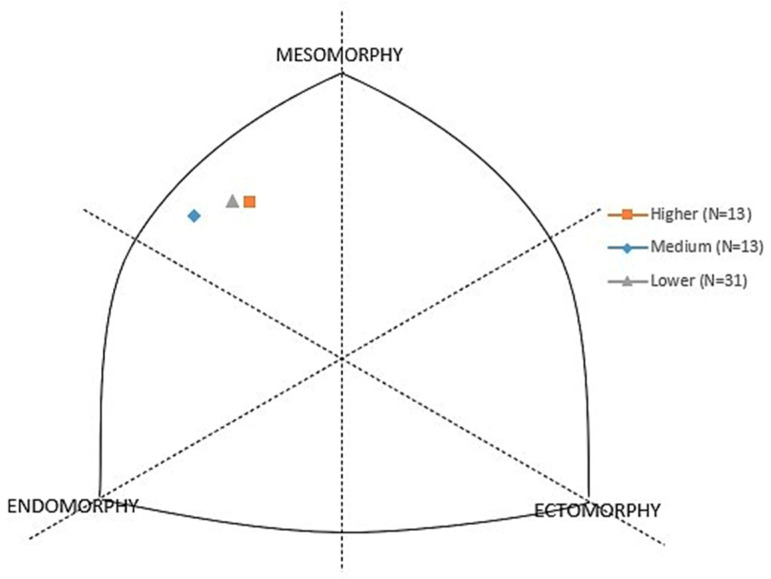
Somatochart.

Figure 1 is graphic representation of the somatotype ratings of Finn class sailors divided into three groups according to their level of general competitive success: *higher level* (square), *medium level* (rhomb), and *lower level* (triangle).

## 4. Discussion

There are several major findings from this study: (a) anthropometric profiles of elite Finn class sailors have been determined; (b) somatotype profiles of elite Finn class sailors have been determined; and (c) significant differences between more-successful, medium, and less-successful sailors in some anthropometric parameters have been found. These findings require a more precise and detailed interpretation and will be further presented.

### 4.1. Finn Sailors Anthropometric Parameters Comparison to Previous Findings

The lack of scientific literature on a sample of elite Finn class sailors limits the possibility of quality comparison of the sample observed in this study to those of other authors. Furthermore, there are other problems: the research includes small subject samples, ranging from three to eight sailors [18,22,23,31,32], a small number of analyzed morphological characteristics [18,22,23,24,25,32], and a period in which the sailors were measured. Finn sailors in the available scientific literature were measured between 1995 and 2018, which would not be a problem if two new rules had not been adopted in this period that could affect sailors’ morphological characteristics. The first rule was adopted in 1995 and it refers to prohibiting the use of the “weight jacket”; the second rule was adopted in 2000 and it refers to permitting pumping when sailing downwind.

Average body mass values in the observed sample of Finn sailors are ±1 kg compared to the body mass values recorded in other studies [22,24,32].

Finn sailors were from 8.6 kg to 7.4 kg lighter [18,23] before 1995, when the “weight jackets” were banned from sailing. By using the “weight jacket”, a sailor could add weight to achieve better momentum in straightening the boat when sailing upwind, which ultimately allowed for greater speed in strong-wind conditions. On the other hand, in low-wind conditions, a sailor with lower body mass and without the “weight jacket” might be more agile and mobile when sailing and maneuvering. Furthermore, the positive impact of the reduced overall weight of the sailboat on the reduction in hydrodynamic resistance at lower speeds is also not negligible. Depending on the speed of the wind and the sailor’s body mass, the “weight jacket” mass could range from 0.5 kg to 10 kg.

Pezelj et al. [15] also recorded lower body mass values. In their study, the body mass of U23 Finn sailors whose average age was 20.8 ± 1.27 years was 92.07 ± 5.66 kg. We may also interpret this difference in body mass by the fact that the subjects in the study conducted by Pezelj et al. [15] were, on average, 5 years younger than the observed sample and have not yet reached the “optimum” body mass for sailing in the Finn class.

Maiseti et al. [24] and Sanchez and Banos [31] recorded higher average body mass values of Finn sailors as compared to the observed sample. Finn sailors (n = 24) who participated in the 2000 Olympics had an average body mass of 97.5 ± 7.5 kg [24], whereas the members of the Spanish pre-Olympic team (n = 4) had an average body mass of 99.1 ± 7.3 kg [31].

We should take the comparison of body fat percentage between Finn sailors in the observed sample and those in studies conducted by other authors with reservations, considering that the methods of calculation in these studies are not identical. Cunningham [22] determined the body fat percentage of 18.6 ± 3.0% in Finn sailors (N = 8) by using the Durnin and Womersley method, whereas Sanchez and Banos [31] recorded 17.2 ± 2.7% (n = 4) by using the Carter method, and Pezelj et al. [15] used the bioelectric impedance method and recorded a body fat percentage of 13.01 ± 4.02%.

The average stature values in the observed sample of Finn sailors are ±1.5 cm as compared to the stature values determined in other studies [15,18,22,23,24,31,32].

Maiseti et al. [24] recorded 2.5 cm higher values of stature, whereas Bojsen et al. [18] recorded 3.4 cm lower average values of Finn sailors’ stature as compared to the sample in our study.

### 4.2. Finn Sailor Anthropometric Parameters Comparison to Other Sailing Class Sailors Parameters

Comparing the anthropometric characteristics of Finn sailors to sailors in other Olympic classes, it is obvious why it is called the “heavy dinghy” class; in Finn sailors, almost all morphological characteristics have higher average values when compared to the sailors in other Olympic classes [11,17,20,21,23,24,26,32,33]. Average values of body height for Laser sailors recorded in the scientific literature range from 1.724 ± 0.64 m to 1.83 ± 0.3 m, whereas the values of body mass range from 75.6 ± 3.7 kg to 80.6 ± 2.8 kg [11,17,21,23,24,26,31,32,33]. In studies [11,26,31,33] on samples of Laser sailors, the authors have recorded a body fat percentage from 10.5 ± 4.1% to 23.2 ± 12.1%. However, these results should be taken with reservations due to different methods of calculation.

The differences in morphological characteristics of Finn sailors are even more evident when compared to those of elite sailors in two-person Olympic classes: 470 and 49er. The average values of body height for sailors in these classes range from 1.75 m to 1.85 m, whereas their average body mass values range from 61.8 kg to 80.1 kg [31,32]. With their morphological characteristics, Olympic windsurfers in the RSX class can also fit into the stature and body mass range recorded in Laser, 470, and 49er sailors. In studies employing a sample of elite RSX sailors, the authors recorded average body height and body mass values of 1.78 ± 0.05 m and 75.4 ± 3.7 kg, respectively [20], and 1.79 ± 0.02 m and 72.9 ± 2.2 kg, respectively, with an average body fat percentage of 9.8 ± 1% [31].

### 4.3. Somatotype of Finn Sailors

Researchers have identified the *mesomorphic* somatotype component as the dominant component in all elite sailors sailing in Olympic and non-Olympic sailing classes [12,31,33]—even in young sailors in the Optimist class [14]. Elite Finn sailors are no exception. In this study, considering the average values of somatotype categories, it was determined that elite Finn sailors fit the *endomorphic mesomorph* somatotype category. Sailors in the men’s one-person dinghy Olympic classes Laser and Finn [31,33] fit the same somatotype category, whereas sailors in the Olympic classes RSX and 470 fit the *ectomorphic mesomorph* category, with *balanced mesomorph* associated with the 49er class [31].

By analyzing the available literature, it can be noticed that the mesomorphic somatotype component is also the dominant somatotype component in elite athletes in many other Olympic disciplines, e.g., rowing [6], basketball [16], 100 m sprint [5], *off-road* cycling [8]; whereas Finn sailors share the *endomorphic mesomorph* somatotype category with kayakers [16] and rowers [7], and their average values of somatotype components are very similar to those recorded in water polo players of the Spanish national team [10].

### 4.4. Differences between Groups of Finn Sailors According to the General Sailing Achievement Level

For the best possible ranking in the World Sailing Rankings, which was used in this study to define general competitive success, a long-standing continuous participation in as many World Cup Regattas as possible is required. Young, non-established Finn sailors often lack the financial resources for participation in World Cup Regattas around the world; rather, they plan their regatta season more carefully by participating in lower-ranked regional competitions which may bring less points in the WSR rankings. Furthermore, it happens very often that elite sailor in the Laser class, amongst whom are Olympic champions as well, decide to change their sailing class and start competing in the Finn class. Considering that WSR rankings for the Laser and the Finn class are not connected, regardless of their number of points and ranking in the WSR rankings for the Laser class, sailors start their ranking in the Finn class competition with no points.

Through univariate analysis of difference and post hoc analysis, significant differences between more-successful, medium, and less-successful sailors in the variables *age*, *body mass*, *muscle mass*, *arm muscle mass*, and *endomorphy* rating were found.

These results indicate the possibility of selection processes and/or adaptation to sailing occurring in the Finn class. The homogeneity of Finn sailors in the parameters of longitudinal and transverse skeletal dimensions might reflect the selection process, whereas the determined impact and analyses of differences in the dimensions of soft tissue might reflect the adaptive process.

The development process of an elite sailor usually implies going through several sailing classes during his or her career. This transition from class to class follows the sailor’s body growth and development to allow him or her to compete in the sailing class most suited to his or her morphological characteristics. In men’s one-person dinghy, in most cases, this “journey” starts in the Optimist class, across the so-called transition classes, i.e., Laser 4.7 and Laser Radial, to the Olympic Laser Standard and Finn classes. When the height growth decelerates, i.e., stops around the age of 18, sailors start sailing in the Olympic classes. The class selection depends, among other things, upon morphological characteristics, and based on our results, we may conclude that even though they is not yet scientifically determined, the optimal values of body height and other longitudinal and transverse morphological characteristics required for successful sailing in the Finn class are defined quite clearly. Muscle mass and fat mass are morphological characteristics that can be changed by training operators. Thus, findings on the optimal values of soft tissue dimensions required for successful sailing in the Finn class, as well as their impact on competitive success, are extremely important to coaches and sailors. Sailors ranked among the first 40 competitors on the WSR rankings are approximately 5 years older, 3.5 kg heavier, and have greater muscle mass compared to the sailors with a lower ranking. Even though it is not possible to determine whether this is due to sailing in the Finn class or to some other training operators, it can be concluded that older and more-successful sailors have reached optimal values of body mass and muscle mass, whereas younger and less-successful sailors are yet to go through the period of body adaptation.

### 4.5. Limitations of the Study

In this study, only anthropometric parameters were measured. The analysis of morphological variables can only assume the influence of functional and motor abilities on competitive performance, but for a better and more complete analysis of competitive performance in sailing, in addition to morphological characteristics, it is necessary to carry out tests that assess the level of functional and motor abilities. It is unlikely that coaches and athletes would agree on such “stressful” tests prior the major competition, but scientist, coaches, athletes, and class leaders should look for such an option as the findings of such a study could have strong implications for the development of a specific sailing class and for the sailing sport in general.

The WSR is a measure of general sailing performance that expresses a summarized two-year general measure of performance in the Finn class. It is less dependent on, or influenced by, the situational conditions of sailing for this one regatta and the sailing conditions, like wind speed, water conditions, and other environmental factors, which were dominant in that racing week. But it would also be essential to establish differences in morphological parameters between different levels of situational competitive success. The study in question would be affected by all previously mentioned environmental factors, and the results could be interpreted considering wind speed and other sail racing parameters.

Even in this study, a couple of differences among international-level sailors were determined. Studies in which it could be possible to compare club-, national-, and international-level sailors would be beneficial for comparison with the “ideal” Finn class morphological profile. A wider performance range of sailors and possibly a larger number of participants could lead to more morphologically diverse groups; thus, the results might have different implications.

In this study, only univariate analysis was used, providing clear and understandable results and making it suitable for the wider sailing public. For future research, it would be advisable to use multivariate statistical analyses as discriminant analysis or multiple linear regression as it could provide more complex scientific information, especially at the level of latent anthropometric structures.

Future studies could use more demographic data like number of years of sailing in specific sailing class, first sailing class, age when started with sailing, previous sport, etc., and/or some non-sailing sport information pertaining to fitness training as all that information could be related to morphological status and anatomic adaptation to sailing in specific sailing class.

### 4.6. Possible Practical Applications

The values of anthropometric parameters determined for the sample of top Finn sailors may be of great help to coaches and young sailors when deciding on the selection of a senior Olympic class. If the sailors are already actively competing in the Finn class, they can compare their anthropometric characteristics to those of world-elite Finn sailors quite easily and possibly correct those parameters that can be changed under the influence of training, such as muscle mass and fat mass, i.e., body mass in total.

By comparing the anthropometric parameters of Finn sailors with those of sailors in other Olympic classes, it can be concluded that the elimination of the Finn class from the Olympic program could leave elite athletes with these anthropometric characteristics without the possibility of achieving top sports results in Olympic sailing. Thus, this article may serve as an argument in favor of making the decision to reinstate the Finn class in the Olympic program because the anthropologically “heavy-weight” sailors cannot compete successfully in any other current Olympic class.

To the best of our knowledge, this is the first study to determine the anthropometric and somatotype profile of elite Finn sailors or elite sailors in any other Olympic class. In the scientific literature, the impact of morphological characteristics on the general and situational competitive efficacy of elite athletes has been determined in different sports [5,7,8,11,12,13,14,15,16], and this study was conducted in an area of sport which has not yet been investigated—Finn sailing. The subject sample employed in this study does not only represent the sample in this particular population but also the majority of the population of world elite Finn sailors given that the study was conducted just before one of the biggest and most important competitions in the Finn class in a competitive season.

Sailing is sport that includes many sailing classes, of which four are part of the male Olympic sailing program. It would be essential for young sailors to establish and compare the anthropometric and somatotype profiles of elite sailors in each sailing class.

## 5. Conclusions

The anthropometric and somatotype profiles of elite Finn class sailors have been determined. This is the first time such a study has been conducted on sailors in any Olympic sailing class. Differences between groups of Finn sailors—grouped according to the level of general success—in some anthropometric parameters were determined. Anthropometric parameters, e.g., *body mass* and *muscle mass*, are clearly related to sailing performance and efficiency. Choosing a sailing class that is going to match the sailor’s anthropometric profile is one of the most difficult issues for young sailors, so determining the relevant anthropometric parameters of each sailing class could be one of the most important scientific goals in the sailing field. Future research could focus on analyzing the differences among the sailors with respect to situational competitive success. Longitudinal studies could be beneficial to determine the process of anatomic adaptation in sailing at each sailing class.

## Figures and Tables

**Table 1:** Descriptive statistics of anthropometric and somatotype variables of Finn sailors (N = 57).

Variables	Mean ± SD	M	Min	Max	Skew	Kurt	MaxD
Age (yrs)	25.54 ± 4.64	24.96	17.95	41.07	0.90	1.04	0.08
Stature (m)	1.88 ± 0.05	1.87	1.76	2.00	0.35	0.21	0.09
Sitting height (m)	0.98 ± 0.03	0.98	0.89	1.05	−0.53	0.98	0.07
Body mass (kg)	95.17 ± 5.03	95.40	76.30	106.80	−0.85	3.00	0.10
Body mass index (kg/m^2^)	27.07 ± 1.76	27.13	23.17	31.71	0.09	0.13	0.06
Fat range (%)	14.29 ± 3.60	14.20	6.50	20.90	−0.26	−0.56	0.09
Muscle mass (kg)	77.73 ± 4.24	77.90	64.10	90.60	−0.07	1.93	0.07
Trunk muscle mass (kg)	41.67 ± 2.81	41.30	34.00	49.60	0.37	0.83	0.11
Arms muscle mass (kg)	10.14 ± 0.72	10.20	8.50	11.50	−0.10	−0.42	0.07
Legs muscle mass (kg)	25.91 ± 1.34	25.80	21.50	30.10	−0.12	2.33	0.09
Fat mass (kg)	13.62 ± 3.72	13.60	5.70	21.30	−0.09	−0.52	0.06
Trunk fat mass (kg)	7.40 ± 2.53	7.50	1.70	11.90	−0.29	−0.50	0.08
Arms fat mass (kg)	1.51 ± 0.39	1.40	0.80	2.80	0.74	1.54	0.12
Legs fat mass (kg)	4.79 ± 1.22	4.70	2.60	10.20	1.40	5.61	0.09
Biepicondilar humerus width (cm)	7.22 ± 0.40	7.15	6.35	8.15	0.12	−0.37	0.08
Biepicondilar femur width (cm)	9.92 ± 0.57	9.90	8.75	11.40	0.52	0.49	0.08
Upper arm girth flexed and tensed (cm)	38.53 ± 2.13	38.70	32.05	42.85	−0.54	0.37	0.07
Calf girth (cm)	41.06 ± 3.32	41.55	28.20	46.35	−1.62	3.94	0.12
Sum of skinfolds (mm)	57.41 ± 19.14	54.65	24.40	109.75	0.75	0.43	0.10
Triceps skinfold (mm)	12.38 ± 3.92	12.10	5.80	24.05	0.76	1.00	0.10
Subscapular skinfold (mm)	16.58 ± 5.83	15.00	9.20	36.20	1.28	1.62	0.12
Supraspinale skinfold (mm)	16.03 ± 8.88	13.70	5.00	42.20	0.93	0.34	0.13
Medial calf skinfold (mm)	12.42 ± 5.74	11.30	4.40	31.30	1.14	1.25	0.13
Endomorphy rating	3.94 ± 1.19	3.91	1.67	6.73	0.26	−0.51	0.07
Mesomorphy rating	5.50 ± 1.19	5.54	2.10	7.87	−0.42	0.21	0.06
Ectomorphy rating	1.63 ± 0.74	1.52	0.43	3.66	0.79	0.29	0.10

Notes: SD—standard deviation; M—median; Min—minimum result; Max—maximum result; Skew—skewness; Kurt—kurtosis; MaxD—maximum distance between relative cumulative theoretical frequency (normal) and relative cumulative empirical frequency obtained by measuring. The limit value of the KS test for N = 57 is 0.18.

**Table 2:** Analysis of variance (ANOVA) and post hoc analysis between groups of Finn sailors according to their different levels of general success.

Variables	LEVEL OF SUCCESS			ANOVA		Post-hoc LSD Test (between Groups)		
	Higher (N = 13)	Medium (N = 13)	Lower (N = 31)	F	*p*=	*p* = *		
	Mean ± SD	Mean ± SD	Mean ± SD			1–2	1–3	2–3
Age (yrs)	28.74 ± 5.30	27.48 ± 4.39	23.38 ± 3.21	10.07	**0.000**	0.43	**0.000**	**0.003**
Stature (m)	1.89 ± 0.04	1.88 ± 0.05	1.87 ± 0.06	0.53	0.59	0.63	0.31	0.66
Sitting height (m)	0.99 ± 0.03	0.98 ± 0.02	0.98 ± 0.03	0.49	0.61	0.78	0.36	0.55
Body mass (kg)	96.73 ± 3.86	97.49 ± 2.41	93.54 ± 5.73	4.02	**0.02**	0.69	**0.049**	**0.02**
Body mass index (kg/m^2^)	27.18 ± 1.62	27.67 ± 1.17	26.77 ± 1.99	1.23	0.30	0.48	0.49	0.13
Fat range (%)	13.85 ± 3.04	15.48 ± 2.54	13.98 ± 4.15	0.91	0.41	0.26	0.91	0.22
Muscle mass (kg)	79.43 ± 2.91	78.59 ± 2.83	76.65 ± 4.92	2.44	0.10	0.61	**0.047**	0.16
Trunk muscle mass (kg)	42.76 ± 2.28	42.05 ± 1.90	41.05 ± 3.20	1.91	0.16	0.52	0.07	0.28
Arms muscle mass (kg)	10.35 ± 0.63	10.43 ± 0.74	9.94 ± 0.70	2.98	0.06	0.76	0.08	**0.04**
Legs muscle mass (kg)	26.32 ± 1.06	26.11 ± 0.98	25.66 ± 1.54	1.32	0.28	0.68	0.14	0.31
Fat mass (kg)	13.47 ± 3.28	15.11 ± 2.52	13.06 ± 4.20	1.42	0.25	0.26	0.74	0.09
Trunk fat mass (kg)	7.28 ± 2.41	8.33 ± 1.90	7.08 ± 2.76	1.17	0.32	0.29	0.80	0.13
Arms fat mass (kg)	1.52 ± 0.43	1.58 ± 0.22	1.47 ± 0.44	0.36	0.70	0.66	0.75	0.40
Legs fat mass (kg)	4.72 ± 0.66	5.22 ± 0.68	4.63 ± 1.52	1.10	0.34	0.29	0.84	0.15
Biepicondilar humerus width (cm)	7.28 ± 0.29	7.12 ± 0.29	7.23 ± 0.47	0.52	0.60	0.33	0.73	0.42
Biepicondilar femur width (cm)	9.72 ± 0.43	10.00 ± 0.44	9.98 ± 0.66	1.11	0.34	0.22	0.17	0.93
Upper arm girth flexed and tensed (cm)	38.44 ± 1.65	38.96 ± 1.63	38.39 ± 2.49	0.33	0.72	0.54	0.94	0.42
Calf girth (cm)	40.93 ± 3.76	40.60 ± 4.14	41.31 ± 2.82	0.21	0.81	0.81	0.74	0.53
Sum of skinfolds (mm)	51.25 ± 21.45	63.13 ± 15.74	57.61 ± 19.23	1.27	0.29	0.12	0.32	0.38
Triceps skinfold (mm)	10.39 ± 3.02	13.30 ± 2.61	12.83 ± 4.47	2.34	0.11	0.058	0.059	0.71
Subscapular skinfold (mm)	14.79 ± 5.14	17.73 ± 4.80	16.85 ± 6.45	0.90	0.41	0.20	0.29	0.65
Supraspinale skinfold (mm)	14.16 ± 8.67	19.75 ± 9.82	15.26 ± 8.39	1.57	0.22	0.11	0.71	0.13
Medial calf skinfold (mm)	11.90 ± 8.22	12.35 ± 4.13	12.67 ± 5.25	0.08	0.92	0.85	0.70	0.87
Endomorphy rating	3.46 ± 1.22	4.40 ± 1.04	3.94 ± 1.19	2.09	0.13	**0.046**	0.22	0.24
Mesomorphy rating	5.28 ± 1.06	5.43 ± 1.38	5.62 ± 1.19	0.39	0.68	0.75	0.40	0.64
Ectomorphy rating	1.61 ± 0.69	1.42 ± 0.59	1.73 ± 0.82	0.83	0.44	0.50	0.64	0.21

Notes: SD—standard deviation; F—analysis of variance coefficient; *p* = —level of statistical significance; *p* = *—level of statistical significance of Fisher LSD post hoc test between groups of Finn sailors according to their different levels of general success (1—higher; 2—medium; 3—lower).

**Table 3:** Frequency and ratio of somatotype categories of Finn sailors (N = 57).

Somatotype Categories	Frequency	Ratio (%)
Central	2	3.51
Balanced endomorph	1	1.75
Mesomorphic endomorph	2	3.51
Mesomorph–endomorph	6	10.53
Endomorphic mesomorph	39	68.42
Balanced mesomorph	5	8.77
Ectomorphic mesomorph	2	3.51

## Data Availability

The data presented in this study are available on request from the corresponding authors.
